# Evidence-based clinical practice guidelines for chronic pancreatitis 2021

**DOI:** 10.1007/s00535-022-01911-6

**Published:** 2022-08-22

**Authors:** Kyoko Shimizu, Tetsuhide Ito, Atsushi Irisawa, Takao Ohtsuka, Hirotaka Ohara, Atsushi Kanno, Mitsuhiro Kida, Junichi Sakagami, Naohiro Sata, Yoshifumi Takeyama, Junko Tahara, Morihisa Hirota, Nao Fujimori, Atsushi Masamune, Satoshi Mochida, Nobuyuki Enomoto, Tooru Shimosegawa, Kazuhiko Koike

**Affiliations:** 1 Guidelines Committee for Creating and Evaluating the “Evidence-Based Clinical Practice Guidelines for Chronic Pancreatitis’’, The Japanese Society of Gastroenterology, 6F Shimbashi i-MARK Building, 2-6-2 Shimbashi, Minato-ku, Tokyo, 105-0004 Japan; 2 Department of Gastroenterology, Tokyo Women’s Medical University, 8-1 Kawada-cho, Shinjuku-ku, Tokyo, 162-8666 Japan

**Keywords:** Mechanistic definition, Pathogenic fibro-inflammatory syndrome, Early chronic pancreatitis, 2019 CP clinical diagnostic criteria, Diagnosis, Staging, Endoscopic treatment, Surgical treatment, Prognosis, Pancreatic exocrine insufficiency, Pancreatic enzyme replacement therapy, Pancreatogenic diabetes

## Abstract

**Background:**

Chronic pancreatitis (CP) is defined according to the recently proposed mechanistic definition as a pathological fibro-inflammatory syndrome of the pancreas in individuals with genetic, environmental, and/or other risk factors who develop persistent pathological responses to parenchymal injury or stress.

**Methods:**

The clinical practice guidelines for CP in Japan were revised in 2021 based on the 2019 Japanese clinical diagnostic criteria for CP, which incorporate the concept of a pathogenic fibro-inflammatory syndrome in the pancreas. In this third edition, clinical questions are reclassified into clinical questions, background questions, and future research questions.

**Results:**

Based on analysis of newly accumulated evidence, the strength of evidence and recommendations for each clinical question is described in terms of treatment selection, lifestyle guidance, pain control, treatment of exocrine and endocrine insufficiency, and treatment of complications. A flowchart outlining indications, treatment selection, and policies for cases in which treatment is ineffective is provided. For pain control, pharmacological treatment and the indications and timing for endoscopic and surgical treatment have been updated in the revised edition.

**Conclusions:**

These updated guidelines provide clinicians with useful information to assist in the diagnosis and treatment of CP.

## Introduction

The clinical practice guidelines for chronic pancreatitis (CP) have a history of being prepared in accordance with revisions to the clinical diagnostic criteria for CP. The first edition of the clinical practice guidelines for CP was created in 2009 based on the 2001 clinical diagnostic criteria, and the second edition [1] was published in 2015 with addition of new diagnostic criteria for early stage CP from the 2009 revision of the clinical diagnostic criteria [2]. This third edition, namely, the 2021 clinical practice guidelines for CP, is based on the 2019 revision of the clinical diagnostic criteria for CP [3, 4], which incorporate the concept of a pathogenic fibro-inflammatory syndrome, which involves persistent inflammation and fibrosis of the parenchyma.

The revision work was carried out in accordance with the Minds Guide for the Development of Clinical Practice Guidelines 2017 [5] and the JSGE Clinical Practice Guidelines [6]. The JSGE guidelines committee decided to reclassify the clinical questions into three categories: clinical questions (CQ), the answers to which are associated with important outcomes in the guidelines and were established by an exhaustive literature review; background questions (BQ), which generally have 100% consensus; and future research questions (FRQ) that do not have sufficient data available to be considered as CQ, but are important issues for the future. For CQ and FRQ, the literature search covered the period from 1989 to December 31, 2019, and included both English- and Japanese-language studies. Important new evidence that emerged between the end of the literature search period and June 2021 was added through a manual search. The level of evidence in general was evaluated using the GRADE (The Grading of Recommendations Assessment Development and Evaluation) system [7]. The strength of each recommendation was based on (1) certainty of evidence (strength), (2) patient preferences, (3) benefits and harms, and (4) cost evaluation. The quality of the evidence was graded as A (high), B (moderate), C (low), or D (very low).

Experts voted using a modified Delphi method and the nominal group technique, and consensus was deemed to have been reached when at least 70% of experts were in agreement.

## Definition

The concept of a new international mechanistic definition of CP has been proposed [8]. According to this definition, CP is defined as a pathogenic fibro-inflammatory syndrome of the pancreas in which a persistent pathological response to pancreatic parenchymal injury or stress occurs in individuals with genetic, environmental, and/or other risk factors. In Japan, diagnosis of CP is based on the 2019 clinical diagnostic criteria [3, 4]. According to this new concept, patients with CP are considered to be at risk or to have acute pancreatitis-recurrent acute pancreatitis, early CP, established CP, or end-stage CP. Thus, CP based on the 2009 clinical diagnostic criteria [2] corresponds to established CP and end-stage CP in this conceptual model. Early CP has not reached established CP, and it is assumed that some biomarkers and pathological changes that reflect the pathophysiology of CP will be detected to distinguish it from acute pancreatitis-recurrent acute pancreatitis.

## Diagnosis

**Fig. 1:**
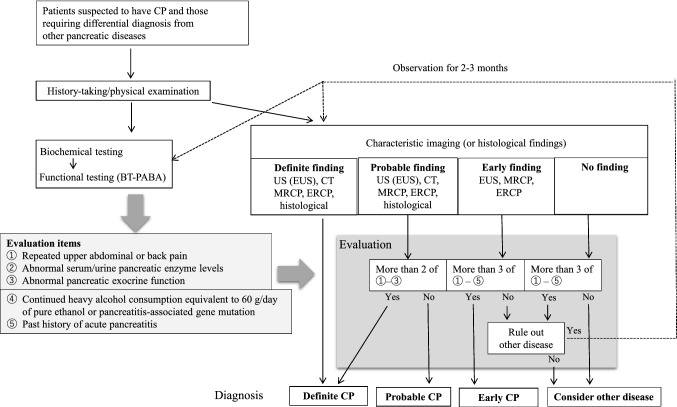
Diagnostic pathway for chronic pancreatitis. *Careful follow-up is required for a suspected case of early CP when two items of (1) to (5) are positive and other diseases are excluded in cases showing imaging findings that suggest early CP. *BT-PABA* pancreatic function diagnostant test, *CT* computed tomography, *CP* chronic pancreatitis, *ERCP* endoscopic retrograde cholangiopancreatography, *EUS* endoscopic ultrasound, *MRCP* magnetic resonance cholangiopancreatography, *US* ultrasonography

When CP is suspected, a diagnosis should be made according to the 2019 clinical diagnostic criteria for CP [3, 4]. The 2009 clinical diagnostic criteria for CP [2] introduced the concept of early CP for the first time, and were revised in 2019 with the intention of implementing diagnostic criteria with higher specificity based on the findings of a nationwide epidemiologic survey of early CP [9] and a prospective prognosis survey [10]. A flowchart showing the process for differential diagnosis of CP is provided in Fig. 1. Diagnostic elements consist of characteristic imaging findings, characteristic histological findings, and five evaluation elements. Patients are diagnosed as having early CP if they have three or more of the five evaluation items and findings characteristic of early CP on endoscopic ultrasound (EUS), endoscopic retrograde cholangiopancreatography (ERCP), or magnetic resonance cholangiopancreatography (MRCP).

Abdominal ultrasound is useful for diagnosis of stones, dilation of the pancreatic duct, and pancreatic atrophy, but these features are often difficult to observe if the patient is in poor physical condition. A systematic review [11] found that abdominal ultrasound had sensitivity of 67% [95% confidence interval (CI) 53–78%], which is clearly lower than that for other diagnostic imaging methods. A study in the United States found that when the findings in the pancreatic duct and parenchyma were assessed according to the Rosemont classification [12], which is a system of criteria for diagnosing CP by EUS, the sensitivity increased to 81% and the specificity increased to 97% [13].

Computed tomography (CT) is useful for diagnosing CP, but it is difficult to diagnose early CP using CT. Despite having inferior diagnostic ability to that of EUS, abdominal CT has been reported to be useful for diagnosis of CP [14–16]. In a systematic review of the various diagnostic imaging methods used for CP, abdominal CT had sensitivity of 75% and specificity of 91%, indicating excellent diagnostic ability [11]. When early stage CP is suspected, it is difficult to detect subtle changes in the pancreatic parenchyma by CT (9), and evaluation by EUS should be added [3, 4].

### CQ: is abdominal magnetic resonance imaging/MRCP recommended for diagnosis of CP?

- Abdominal magnetic resonance imaging/MRCP is useful for the diagnosis of CP, and we suggest that it be performed.

Strength of recommendation: weak, evidence level: B

MRCP has been shown to have high diagnostic ability for CP and a good diagnostic correlation with ERCP [17, 18]. In a recent meta-analysis, the diagnostic ability of MRCP for CP had sensitivity of 78% and specificity of 96%, which are almost the same as those of ERCP [11]. Recently, secretin-stimulated MRCP has been shown to have high diagnostic ability for early CP and evaluation of exocrine function [19–21]. Although ERCP is useful for diagnosing CP, it should be performed after carefully considering the indications.

### CQ: is endoscopic ultrasound recommended for diagnosis of CP?

- EUS is useful for diagnosing CP/early CP, because it allows detailed observation of the pancreatic parenchyma and the morphology of the pancreatic duct, and has been proposed as treatment.

Strength of recommendation: weak, evidence level: B

The 2009 clinical diagnostic criteria for CP [2] include seven EUS findings for diagnosis of early CP with reference to the Rosemont classification (12). The validity of each EUS diagnostic element in the examination has also been demonstrated from the standpoint of risk factors for CP [22, 23]. A multicenter prospective study that sought to confirm the validity of these diagnostic criteria found that about 5% of cases diagnosed as early CP by this criteria progressed to definite or probable CP [10]. In the 2019 clinical diagnostic criteria for CP [3], similar findings for early CP were integrated and the number of items was reduced from seven to four to increase the specificity of EUS diagnosis: (1) hyperechoic foci (non-shadowing) or strands, (2) lobularity, (3) a hyperechoic main pancreatic duct margin, and (4) dilated side branches. At least two of these four EUS findings, including (1) or (2), are required for a diagnosis of early CP. In recent years, pancreatic hardness measured by EUS elastography has been investigated for its ability to diagnose early stage CP [24].

## Etiology

### FRQ: which patients should be tested for pancreatitis-associated genetic abnormalities?

- A search for *PRSS1* and *SPINK1* gene abnormalities should be considered in patients with juvenile pancreatitis or CP of unknown origin and a family history.
- It is hoped that greater consensus will be reached on the genes to be analyzed, gene counseling for mutation-positive individuals, and pancreatic cancer screening methods in patients with hereditary pancreatitis.

Since the report identifying mutation of the cationic trypsinogen (*PRSS1*) gene as the cause of hereditary pancreatitis in 1996 [25], abnormalities in various pancreatitis-associated genes, including cystic fibrosis membrane conductance regulator (*CFTR*) [26, 27], pancreatic secretory trypsin inhibitor (*SPINK1*) [28], chymotrypsin C (*CTRC*) [29], carboxypeptidase A1 (*CPA1*) [30], and calcium ion channel *TRPV6* [31], have been reported. In the Japan National Survey of Hereditary Pancreatitis [32], mutations in *PRSS1* were found in 30 of 73 families (41.1%) and mutations in *SPINK1* in 26 (35.6%). Genetic testing for *PRSS1* and *SPINK1* in patients with hereditary pancreatitis has been reported in Europe and the US [33, 34]. Furthermore, the American College of Gastroenterology guideline for CP [35] recommends that genetic tests for *PRSS1*, *SPINK1*, *CFTR*, and *CTRC* be performed for CP of unknown origin, especially in young patients. In the 2019 clinical diagnostic criteria for CP [3, 4], mutations in established pancreatitis-associated genes, such as *PRSS1* and *SPINK1*, are included in the diagnostic items for early stage CP, and the role of genetic testing in daily clinical practice is increasing. However, pancreatitis-associated genetic tests are currently not covered by national health insurance in Japan, and it is still unclear how abnormalities in *SPINK1* should be handled in the diagnostic criteria for hereditary pancreatitis. It is hoped that greater consensus will be reached by combining the genes to be analyzed in the same genetic test, genetic counseling for mutation-positive individuals, and improved screening methods for pancreatic cancer in patients with hereditary pancreatitis.

## Staging

CP is classified into latent, compensatory, transitional, and decompensated stages according to the degree of pancreatic endocrine and exocrine dysfunction.

Abdominal pain is the main symptom during the latent to compensatory stage, when there is no obvious impairment of pancreatic endocrine and exocrine function. In the decompensated stage, pancreatic endocrine and exocrine dysfunction becomes the main symptom. Pancreatic exocrine dysfunction is caused by a deficiency in pancreatic enzymes and manifests as digestive and absorptive disorders. Patients with pancreatic exocrine dysfunction are susceptible to malnutrition because of impaired digestion and absorption of lipids and caloric loss as a result of increased fat excretion in the stool. Pancreatic exocrine dysfunction also causes various symptoms, including steatorrhea, abdominal distension, and deficiencies in fat-soluble vitamins (A, D, E, and K) and essential fatty acids, which reduce the patient’s quality of life [36, 37]. Moreover, pancreatic exocrine insufficiency has recently been reported to be associated with a decrease in muscle mass (sarcopenia), which leads to a decrease in muscle strength and impaired physical function [38]. There are two types of pancreatic exocrine function tests: a direct method for evaluating pancreatic exocrine function under secretin stimulation and an indirect method under non-stimulation. Indirect pancreatic exocrine function tests include measurement of fecal chymotrypsin activity, measurement of fecal elastase 1, the ^13^C-dipeptide (benzoyl-L-tyrosyl-[1-^13^C] alanine: Bz-Tyr-Ala) breath test, the BT-PABA (pancreatic function diagnostant) test, and cine-dynamic MRCP. A diagnosis of exocrine pancreatic insufficiency is based on clinical signs, nutritional index markers, and the BT-PABA test. The fecal elastase 1, the ^13^C-labeled breath tests, and cine-dynamic MRCP are currently not covered by national health insurance in Japan.

Pancreatic endocrine dysfunction is positioned as diabetes mellitus secondary to pancreatic disease, such as CP and pancreatic cancer, or following pancreatectomy. Glycemic control is often unstable in these patients because of reduced secretion of insulin and glucagon.

## Treatment

**Fig. 2:**
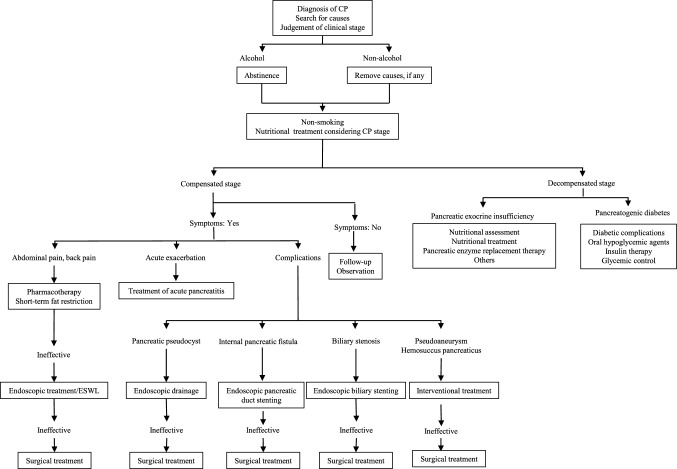
Therapeutic pathway for chronic pancreatitis. *CP* chronic pancreatitis, *ESWL* extracorporeal shock wave lithotripsy

Treatment strategies include pharmacological agents, nutritional therapy, lifestyle guidance, endoscopic treatment, and surgery depending on symptoms, pancreatic exocrine and endocrine function, and various complications. A flowchart summarizing the treatment options is shown in Fig. 2.

Alcohol is a risk factor for progression from acute alcoholic pancreatitis to CP, and abstinence may prevent recurrent acute pancreatitis and progression to CP [39, 40]. According to a survey of the prognosis of acute pancreatitis in Japan, the percentage of patients who progressed to CP after acute alcoholic pancreatitis was 13.6% in cases of complete abstinence, 23.3% in cases of limited alcohol consumption but still drinking daily, and 40.9% when alcohol consumption remained unchanged [41]. Abstinence is important for improvement of the prognosis in patients with alcoholic CP. In a study of alcohol abstinence in patients with acute alcoholic pancreatitis, the recurrence rate was significantly lower in patients who were repeatedly instructed to abstain from alcohol after discharge than in those who were instructed to abstain from alcohol only once at the time of discharge [42]. In principle, patients with alcoholic pancreatitis should be instructed to refrain from alcohol, which means permanent abstinence. Abstinence from smoking may also prevent progression of CP; therefore, the recommendation is to provide guidance on smoking cessation.

**Fig. 3:**
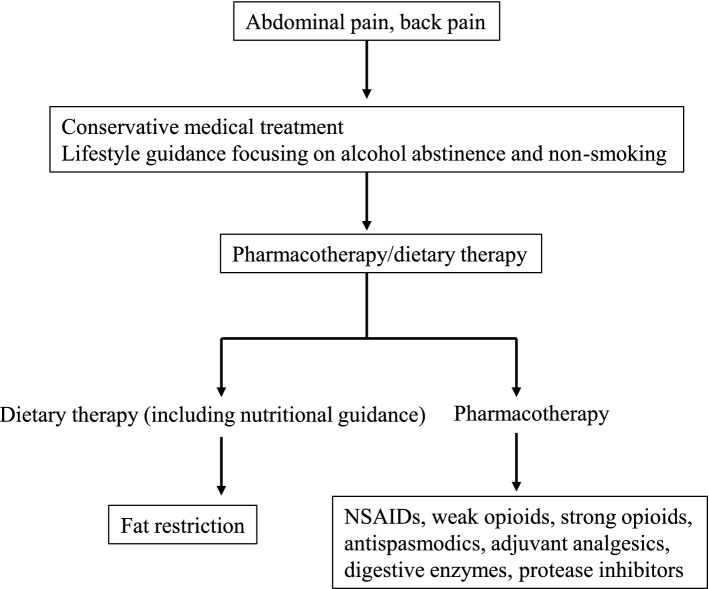
Conservative medical treatment pathway for chronic pancreatitis. In cases of acute exacerbation, the severity of acute pancreatitis and the treatment strategy should be determined promptly. Treatment with an elemental diet may be considered. Choice of pharmacotherapy and selection of the dose should be decided in reference to this figure

In the compensated stage, prevention of repeated relapses and pain takes priority. Nutritional therapy appropriate to the disease stage is useful for patients with CP. A fat-restricted diet is effective for patients in the compensatory stage who have abdominal pain. Nonsteroidal anti-inflammatory drugs (NSAIDs) are the first choice for analgesia, and if inadequate, weak opioids are useful for both abdominal pain and back pain. If analgesia is still inadequate, strong opioids may be used (see Fig. 3). Endoscopic treatment is used in patients with abdominal pain resulting from obstruction of the pancreatic duct. Extracorporeal shock wave lithotripsy (ESWL), endoscopic treatment, and surgery are options for treatment of pancreatic stones. Endoscopic treatment includes endoscopic pancreatic duct incision, endoscopic stone removal, and endoscopic placement of a pancreatic duct stent. For stenosis/occlusion of the pancreatic duct, plastic stents, metallic stents, and dumbbell-type stents with a terminal shape that is less likely to cause stent invasion or stent-induced pancreatic duct stenosis can be placed. Transpapillary drainage or EUS (transgastrointestinal, antegrade, and rendezvous) drainage of pseudocysts due to CP is recommended. If endoscopic drainage is difficult, surgery should be considered. EUS/CT-guided celiac plexus block/neurolysis is effective in the short term for abdominal pain, but is less effective in the long term. Interventional radiology is useful for pseudoaneurysm and hemosuccus pancreaticus associated with CP.

In the decompensated stage, treatment is required for digestive malabsorption, nutritional disorders, and diabetes mellitus caused by decreased pancreatic endocrine and exocrine function. Patients with exocrine pancreatic insufficiency should receive pancreatic enzyme replacement therapy and adequate nutrition without fat restriction. Fat-soluble vitamin (A, D, E, and K) supplementation can be given after pancreatic enzyme replacement therapy depending on the degree of exocrine pancreatic insufficiency and nutritional status. Prospective observational studies have shown that patients with CP have decreased bone mineral density [43]. It has been reported that 23.4% (95% CI 16.6–32.0) of patients with CP have osteoporosis and 39.8% (95% CI 29.1–51.6) have osteopenia [44]. Cross-sectional observational studies have also found that bone mineral density decreases in patients with CP regardless of its cause and duration [45]. The decrease in bone density and bone disease caused by CP are now known as CP-associated osteopathy [44]. Sarcopenia has been reported to lead to a significantly increased hospitalization risk and mortality risk in patients with CP [46], and pancreatic exocrine dysfunction is an independent risk factor for sarcopenia [38, 46]. Although there is not a high level of evidence from studies of treatment methods for CP-associated sarcopenia, nutritional therapy, including appropriate pancreatic enzyme replacement therapy for pancreatic exocrine dysfunction, is presumed to be useful [47].

### CQ: is smoking cessation guidance recommended for treatment of CP?

- Smoking cessation guidance is recommended for treatment of CP.

Strength of recommendation: strong, evidence level: C

In a meta-analysis of 10 observational studies (*n* = 22,850), smoking significantly increased the risk of developing CP [relative risk (RR) 2.29, 95% CI 2.08–2.51; *p* < 0.00001] [48–57]. Furthermore, smoking was found to promote pancreatic calcification (RR 1.44, 95% CI 1.25–1.67; *p* < 0.00001) [58–64] and to increase the risk of developing pancreatic exocrine dysfunction (RR 1.62, 95% CI 1.29–2.04, *p* < 0.00001) and diabetes (RR 1.28, 95% CI 1.10–1.48; *p* = 0.001) in a meta-analysis of seven observational studies of pancreatic calcification (*n* = 2953), four observational studies of pancreatic exocrine dysfunction (*n* = 1331), and five observational studies of diabetes (*n* = 2254) [58–65]. In a meta-analysis of two observational studies, smoking also worsened the long-term prognosis after endoscopic treatment in patients with painful CP (RR 4.73, 95% CI 2.15–10.40; *p* = 0.0001) [66, 67]. In contrast, a meta-analysis of five observational studies showed that smoking cessation had the effect of suppressing the onset of CP (RR 0.58, 95% CI 0.51–0.67; *p* < 0.00001) [51–55]. A study in which 360 patients with CP underwent long-term follow-up (for a mean of 19 years) found that those who quit smoking within 5 years after onset of CP had a significantly reduced risk of pancreatic calcification in comparison with those who continued to smoke (RR 0.44, 95% CI 0.22–0.87; *p* = 0.02) [62]. A meta-analysis of two observational studies in which ESWL and endoscopic treatment were performed in patients with painful CP who underwent long-term follow-up found that relapse of pain was significantly less likely in those who stopping smoking than in those who continued smoking (RR 0.20, 95% CI 0.08–0.54; *p* = 0.001) [66, 68].

### CQ: are nonopioid analgesics, opioids, and analgesic adjuvants recommended for treatment of pain?

- Weak opioids are recommended if NSAIDs are ineffective.

Strength of recommendation: weak, evidence level: C

- If adequate doses of NSAIDs or weak opioids are ineffective, consider endoscopic treatment or surgery. Strong opioids should be reserved for patients for whom these treatments are not indicated.

Strength of recommendation: weak, evidence level: D

NSAIDs and anticholinergic agents that suppress pancreatic exocrine stimulation via the vagus nerve have been widely used in CP. However, a weak opioid is recommended when NSAIDs are not sufficiently effective. Tramadol is a weak opioid that was demonstrated to have an analgesic effect equivalent to that of morphine in a randomized-controlled trial (RCT) that included patients whose pain did not improve after 2 weeks of an NSAID [69]. Furthermore, compared with morphine, tramadol has fewer psychological and gastrointestinal side effects [70]. A study of the pharmacokinetics of oral and intravenous administration of acetaminophen in patients with CP found a low concentration of acetaminophen in the blood and suggested that administration of additional analgesics should be considered in these patients [71]. The American Gastroenterological Society guidelines on the treatment of pain in CP [72] and the German guidelines for treatment of CP [73] recommend use of NSAIDs and non-narcotic analgesics in stages, and if they are not sufficiently effective, narcotic agents can be used. Recent reports suggest that the analgesic effect of oxycodone may be better than that of morphine because of its kappa agonist activity [71]. An RCT reported a few years ago found that pain associated with CP was alleviated by a combination of antioxidants and pregabalin [74].

### CQ: is pancreatic enzyme replacement therapy recommended for treatment of pain?

- Pancreatic enzyme replacement therapy should not be used to treat pain in patients with CP. However, it may be beneficial for abdominal symptoms, such as abdominal distention and flatulence, associated with pancreatic exocrine dysfunction.

Strength of recommendation: weak, evidence level: C

A meta-analysis [75], systematic review [76], and Cochrane review [77] could not demonstrate the efficacy of pancreatic enzyme replacement therapy for pain in CP. However, its effectiveness for abdominal pain and flatulence due to exocrine pancreatic insufficiency has been confirmed [78–80].

### CQ: are proteolytic enzyme inhibitors recommended for treatment of pain?

- We propose use of a proteolytic enzyme inhibitor as a treatment for pain.

Strength of recommendation: weak, evidence level: D

Proteolytic enzyme inhibitors have an inhibitory effect on trypsin activity and are thought to suppress the progression of pancreatitis by suppressing activation of pancreatic enzymes. Abdominal symptoms were reported to improve in patients with nonalcoholic CP who received proteolytic enzyme inhibitors if they had a higher number of positive EUS findings [81]. A combination of camostat mesilate, pancrelipase, and rabeprazole has been reported to achieve significant improvement in epigastric pain in patients with early stage CP [82].

### CQ: is long-term repeated endoscopic treatment recommended for treatment of pain?

- Long-term repeated endoscopic treatment (for more than 2–3 years) should not be used to treat pain in patients with CP.

Strength of recommendation: weak, evidence level: C

In randomized-controlled trials, pain scores were significantly improved in patients with CP-related pain who underwent surgery first in comparison with those who underwent endoscopic treatment first [83–86]. Furthermore, in one of these studies, the number of treatment procedures was significantly lower in the surgical treatment group [86]. Although the superiority of surgery is recognized, endoscopic treatment is currently positioned as first-line treatment in view of its minimal invasiveness and low complication rate. A systematic review reported that pain severity and reintervention and pancreatic insufficiency rates were lower after early surgical intervention (within 3 years) than after late surgery (3 years and beyond) [87]. Another study found no significant difference in the complete or partial pain disappearance rate between endoscopic treatment (+ ESWL) and surgery in patients with pancreatic stones in the head and body of the pancreas but no inflammatory mass, duodenal stenosis, or biliary stricture [88]. In summary, repeated endoscopic treatment should not be used over the long term (2–3 years) in patients with CP-related pain. However, given that endoscopic treatment is less invasive than surgery and is effective in some cases, endoscopic treatment (+ ESWL) can be performed over a period of 1–2 years. However, the evidence is still inadequate and more studies are needed to clarify the value of repeated endoscopic treatment in the long term.

### CQ: is surgical treatment recommended for treatment of pain when endoscopic treatment is ineffective?

- Surgical treatment is recommended for patients in whom endoscopic treatment has been ineffective for pain relief.

Strength of recommendation: strong, evidence level: B

**Fig. 4:**
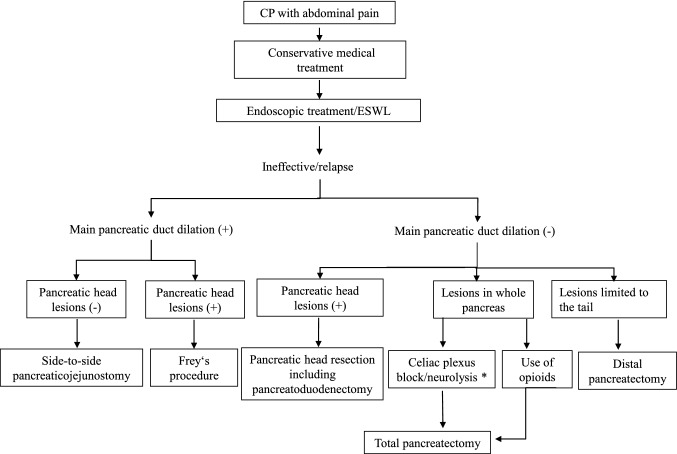
Surgical treatment pathway for chronic pancreatitis. If a malignant tumor cannot be ruled out, pancreaticoduodenectomy is performed for a lesion at the head of the pancreas and distal pancreatectomy with lymph-node dissection for a lesion at the tail of the pancreas. *Effective in the short term but not in the long term. *CP* chronic pancreatitis, *ESWL* extracorporeal shock wave lithotripsy

Endoscopic treatment is unlikely to achieve long-term pain control. In a study of patients in whom pain relapsed during long-term follow-up after insertion of a pancreatic duct stent, the results in terms of severity of pain, weight gain, and reintegration were better in those who underwent surgery than in those who underwent stent reinsertion [89]. Pancreatectomy, pancreaticojejunostomy, and abscess drainage were reported to achieve complete resolution of pain in approximately 62.5% of cases in which pain resolution had been inadequate after endoscopic pancreatic stenting [90], suggesting that surgery can be helpful when endoscopic pancreatic stenting has been ineffective. Endoscopic stenting of the pancreatic duct was reported to have no adverse effects on the outcome of subsequent pancreaticojejunostomy in patients with CP [91]. Furthermore, according to three meta-analyses, the pain remission rate after surgical treatment was 80.4% and significantly higher than that after endoscopic treatment which was 72.6%, whereas the complication rate associated with surgical treatment was 12.7% and that associated with endoscopic treatment was 10.1%. These findings indicate that surgery is superior to endoscopic treatment in terms of pain relief. Furthermore, there is evidence, suggesting that there is no difference in the incidence of complications in patients with CP and pancreatic duct dilation [92–94]. Although endoscopic treatment is the first choice for pain control in patients with CP for whom conservative medical treatment is ineffective, surgical treatment is recommended for the cases in which endoscopic treatment is unsuccessful or ineffective (Fig. 4).

### CQ: is a fat-restricted diet recommended for treatment of pancreatic exocrine insufficiency?

- A uniform fat-restricted diet is not recommended in the decompensated stage of CP with exocrine pancreatic insufficiency.

Strength of recommendation: strong, evidence level: D

A short-term low-fat diet (fat 30–35 g/day; fat ≤ 10 g/meal) is recommended for patients with compensatory abdominal pain and back pain. However, in the decompensated stage with pancreatic exocrine insufficiency, a daily fat intake of 40–70 g or 30%–40% of total calories is recommended in combination with pancreatic enzyme replacement therapy to prevent malnutrition [95]. The basis of treatment in the decompensated phase is both administration of sufficient pancreatic enzyme replacement therapy and an appropriate energy intake. Excessive dietary restrictions, including fat restriction, must be avoided, because they worsen malnutrition [96].

### CQ: is pancrelipase recommended for treatment of pancreatic exocrine insufficiency?

- Pancrelipase is a high-titer pancreatic enzyme preparation that can be recommended for treatment of pancreatic exocrine insufficiency with steatorrhea and weight loss.

Strength of recommendation; strong, evidence level: A

Significant improvements in fat absorption, nitrogen absorption, and fecal fat content have been reported in multiple randomized-controlled trials of pancrelipase in patients with pancreatic insufficiency due to CP or following pancreatic surgery [79, 80, 97]. Furthermore, a multicenter questionnaire-based survey found that pancrelipase improved quality of life, for example, by improving weight loss and steatorrhea [98].

### CQ: are agents that suppress gastric acid recommended for treatment of pancreatic exocrine insufficiency?

- If the therapeutic effect of pancreatic enzyme replacement therapy is inadequate in patients with pancreatic exocrine insufficiency, an H2-receptor antagonist or proton pump inhibitor can be used.

Strength of recommendation: weak, evidence level: C

In cases of pancreatic exocrine insufficiency, the pH in the upper small intestine is lower because of a decreased bicarbonate concentration in pancreatic juice. A pH of < 4 in the small intestine inactivates pancreatic lipase, and precipitation of bile acid leads to poor formation of micelles. Furthermore, enteric-coated digestive enzyme preparations are not released at a pH < 5. Treatment with a proton pump inhibitor or H2-receptor antagonist has been shown to be effective when combined with a pancreatic digestive enzyme agent, whether enteric-coated or not, in patients with steatorrhea [99–101]. Combined use of a gastric acid-suppressing agent has been reported to be effective when the capacity to secrete gastric acid is normal or high [100, 101]. However, the additive effect of a gastric acid-suppressing agent was not confirmed in some studies [102, 103]. Therefore, it is not necessary to consider routine use of a combination of enteric-coated pancreatic digestive enzyme therapy and a gastric acid-suppressing agent. However, it is worth adding an agent that suppresses gastric acid secretion when pancreatic digestive enzyme replacement therapy is not sufficiently effective.

### CQ: is calorie restriction similar to that used in primary diabetes recommended when pancreatic exocrine insufficiency is complicated by diabetes?

- Uniform calorie restriction for pancreatogenic diabetes is not recommended in view of the risk of poor nutrition and hypoglycemia. Glycemic control should be performed in combination with treatment for pancreatic exocrine insufficiency with an appropriate energy intake.

Strength of recommendation: strong, evidence level: D

Pancreatogenic diabetes in the decompensated stage of CP is often associated with exocrine pancreatic insufficiency. It is necessary to evaluate the endocrine and exocrine function of the pancreas and manage nutrition from a long-term perspective. Although there is no explicit treatment policy for calorie intake in patients with diabetes secondary to CP, excessive calorie restriction is not recommended, because it results in poor nutritional status and hypoglycemia [96, 104]. Energy metabolism in patients with pancreatogenic diabetes may be higher than in healthy individuals and should be evaluated on a case-by-case basis. The dietary content, including the carbohydrate and fat intake, should be adjusted with the cooperation of a registered dietitian while monitoring the daily blood glucose level according to the needs of the individual patient [105]. In patients with diabetes secondary to CP and pancreatic exocrine insufficiency, it is important to control blood glucose levels after treating poor digestion and malabsorption and to maintain an appropriate energy intake.

### CQ: are oral hypoglycemic agents recommended for treatment of diabetes secondary to CP?

- Oral hypoglycemic agents are recommended for pancreatogenic diabetes whether the patient has insulin resistance or normal insulin secretory capacity.

Strength of recommendation: weak, evidence level: D

The main treatment for diabetes secondary to CP is insulin, given that the diabetes is caused by insulin deficiency due to depletion of pancreatic β-cells [106]. In the National Epidemiological Survey on Pancreatic Diabetes in Japan, 66.7% of patients with CP-associated diabetes were treated with insulin [107]. However, there are some cases in which the pathophysiology of type 2 diabetes is included as CP-associated diabetes [108]. Although there is not sufficient evidence supporting the efficacy of oral hypoglycemic agents for pancreatogenic diabetes, medication for insulin resistance and insulin secretagogues may be effective if insulin resistance is suspected or insulin secretory capacity is maintained. Metformin has been reported to reduce the risk of developing pancreatic cancer and to improve the prognosis. Although metformin is widely used first-line for CP-associated diabetes [109], there is a report, suggesting that it has no effect on the prognosis [110], and there is little clear evidence to warrant its recommendation at this time.

### CQ: is insulin therapy recommended for treatment of pancreatogenic diabetes mellitus?

- Insulin therapy is recommended for patients with insulin-dependent diabetes mellitus.

Strength of recommendation: strong, evidence level: C

In the National Epidemiological Survey on Pancreatic Diabetes in Japan (2005), 66.7% of respondents were receiving insulin. According to the 2019 diabetes practice guideline [111], insulin therapy is an absolute indication regardless of the type of diabetes if it is insulin-dependent. Considering that endocrine insufficiency in patients with CP is accompanied by deficient glucagon secretion and that hypoglycemia is likely to occur and be prolonged, insulin injections at a frequency close to that of the physiological secretion pattern of insulin are recommended [106, 112].

### CQ: is drainage recommended for pseudocysts associated with CP?

- Endoscopic drainage is recommended as the first choice for symptomatic pseudocysts.

Strength of recommendation: strong, evidence level: C

Pseudocysts measuring < 4 cm in size or confined to the pancreas may disappear and can be followed up without endoscopic drainage if asymptomatic [113, 114]. In symptomatic cases, drainage is indicated regardless of the size of the cyst. Endoscopic drainage is the first choice and surgery is performed in cases where endoscopic treatment is difficult. Percutaneous drainage takes a long time to perform, and should be considered as an emergency evacuation procedure for patients whose general condition is poor [113–115]. In a meta-analysis of studies comparing endoscopic treatment and surgery, the success rate was higher after surgical intervention but without any difference in the treatment-related complication rate or the pseudocyst recurrence rate. However, endoscopic treatment resulted in short hospital stays and low costs [116]. When endoscopic EUS drainage is performed, a double pigtail-type plastic stent is placed and not removed for at least 6 weeks [113]. Metal stents are not recommended as the first choice because of cost [117, 118]. Drainage is less invasive when performed laparoscopically than when performed via open surgery and may contribute to a lower complication rate and shorter hospital stays, but these benefits are yet to be determined [119]. Laparoscopic drainage of pseudocyst is currently not covered by national health insurance in Japan.

### CQ: is a pancreatic duct stent recommended for internal pancreatic fistula?

- Placement of a pancreatic duct stent is recommended as the initial treatment for internal pancreatic fistula.

Strength of recommendation: weak, evidence level: C

Endoscopic insertion of a pancreatic duct stent (± conventional conservative treatment) is recommended as the first choice for treatment of internal pancreatic fistula and surgery for nonresponsive cases after 3–6 weeks of follow-up [120–122]. The results of treatment are improved by inserting a stent beyond the site of disruption/stenosis in the pancreatic duct. Surgery is performed for patients in whom endoscopic and conservative treatment has been ineffective and for those with complications, such as intra-abdominal infection. The main surgical procedures performed for internal pancreatic fistula are pancreatojejunostomy and cystogastrostomy, with pancreatectomy performed in 10–50% of cases [123, 124].

### CQ: is a bile duct stent recommended for biliary stricture associated with CP?

- The recommendation is to insert multiple plastic stents or a fully covered self-expandable metallic stent (FCSEMS) for CP-associated biliary stricture.

Strength of recommendation: weak, evidence level: B

The 2012 European Society for Gastrointestinal Endoscopy (ESGE) guidelines recommended insertion of multiple plastic stents as first-line treatment [125]. However, there is no difference in results between FCSEMS and insertion of multiple plastic stents and few long-term complications [126, 127]. Therefore, the Asia–Pacific consensus guideline in 2017 recommended insertion of a FCSEMS as the first choice [128]. The 2017 ESGE guidelines also recommend insertion of multiple plastic stents and FCSEMS [129]. However, both procedures are difficult to perform, and it is permissible to insert a single plastic stent in an emergency or for a short period of time.

## Prognosis

Causes of death in patients with CP include malignant tumors, pneumonia, infectious diseases, and diabetes and its complications. Long-term treatment and follow-up are needed according to age of onset and whether there are poor prognostic factors such as alcohol consumption and smoking. It is hoped that defining early CP will increase the likelihood of early intervention to prevent progression to irreversible definitive CP. However, there are no reports demonstrating the effectiveness of therapeutic interventions for early CP that target abstinence from alcohol or smoking cessation. Furthermore, there are no reports on medical intervention for early CP with a high level of evidence. A prospective study that includes a large number of cases is needed in the future to confirm the effect of early medical therapy in patients with early CP.

Although CP is a risk factor for pancreatic cancer, no screening test for pancreatic cancer has been established for patients with CP. In a study of 506 Japanese patients who had been diagnosed with CP more than 2 years earlier, 19 (3.7%) developed pancreatic cancer during a median follow-up of 5.6 years (standardized prevalence ratio 11.8). The risk of pancreatic cancer was 0.11 (95% CI 0.0014–0.80) in surgical cases compared with non-surgical cases [130]. Prevention of inflammation by early intervention may theoretically protect against development of pancreatic cancer; however, most of the relevant studies published to date have been retrospective, with variations in the time from the onset to the intervention and in the follow-up period thereafter. According to the international guidelines, prophylactic pancreatectomy can be considered for individuals with hereditary pancreatitis, who have a lifetime risk of developing pancreatic cancer of 40–55%, but is not recommended for patients without hereditary pancreatitis [131].

### CQ: is endoscopic treatment (+ ESWL) recommended for patients with asymptomatic CP?

- Endoscopic treatment (+ ESWL) is not recommended in patients with asymptomatic CP.

Strength of recommendation: weak, evidence level: C

The efficacy, safety, and cost of endoscopic treatment (+ ESWL) to preserve pancreatic function have not been adequately investigated in asymptomatic cases with pancreatic duct stenosis or pancreatic stones. In view of its invasiveness, uniform endoscopic treatment is not recommended for asymptomatic patients [113, 132]. However, when there is no atrophy of the pancreatic parenchyma and there is a disorder of pancreatic juice secretion caused by pancreatic stones, this treatment can be performed in a specialist facility with adequate informed consent.

### CQ: is surgery recommended to prevent progression of CP?

- Given that early surgery after onset of CP can delay progression, the surgical indications and procedures should be decided after a thorough evaluation of symptoms and complications.

Strength of recommendation: weak, evidence level: C

Surgery is performed for CP when non-surgical treatment is ineffective and when pancreatic cancer is suspected. Pancreatic duct drainage or pancreatectomy is selected according to the pathological condition. Furthermore, the course of subsequent pancreatic function differs depending on the stage of CP at which surgery was performed. Therefore, the effect of surgery on progression of CP cannot be described unequivocally. To date, there have been no studies of surgery performed primarily to prevent progression of a pathological condition. Therefore, the effect on progression must be inferred from long-term postoperative analysis of pancreatic endocrine and exocrine function in symptomatic cases.

A randomized-controlled trial in patients with mild-to-moderate CP found that significantly better pancreatic function was maintained in patients who underwent surgery than in those who did not [133]. Intervention within a period of 3 years has also been shown to reduce the risk of developing postoperative endocrine and exocrine dysfunction to a greater extent than interventions after 3 years or longer [87, 134, 135]. Pancreatic duct drainage (e.g., Frey’s procedure) may be effective from the standpoint of surgical invasion and prevention of pathophysiological progression of CP when performed at an early stage after onset [136]. However, prophylactic surgery is rarely performed to prevent progression in asymptomatic patients.

## Appendix

The members of the Guidelines committee who created and evaluated the Japanese Society of Gastroenterology ‘‘Evidence-based clinical practice guidelines for chronic pancreatitis’’ are listed below.

## Creation committee

Chair: Tetsuhide Ito (Neuroendocrine Tumor Centre, Fukuoka Sanno Hospital, International University of Health and Welfare). Vice Chair: Kyoko Shimizu (Department of Gastroenterology, Tokyo Women’s Medical University).

Members: Atsushi Irisawa (Department of Gastroenterology, Dokkyo Medical University School of Medicine), Takao Ohtsuka (Department of Digestive Surgery, Breast and Thyroid Surgery, Kagoshima University Graduate School of Medical and Dental Science), Hirotaka Ohara (Department of Community-Based Medical Education, Nagoya City University Graduate School of Medical Sciences), Atsushi Kanno (Department of Medicine, Division of Gastroenterology, Jichi Medical University), Mitsuhiro Kida (Department of Gastroenterology, Kitasato University School of Medicine), Junichi Sakagami (Fukuchiyama City Hospital), Naohiro Sata (Department of Surgery, Jichi Medical University), Yoshifumi Takeyama (Department of Surgery, Kindai University Faculty of Medicine), Junko Tahara (Department of Gastroenterology, Tokyo Women’s Medical University), Morihisa Hirota (Division of Gastroenterology, Tohoku Medical and Pharmaceutical University), Nao Fujimori (Department of Medicine and Bioregulatory Science, Graduate School of Medical Sciences, Kyushu University), and Atsushi Masamune (Division of Gastroenterology, Tohoku University Graduate School of Medicine).

## Evaluation committee

Chair: Tooru Shimosegawa (South Miyagi Medical Center). Vice Chair: Masanori Sugiyama (Tokyo Rosai Hospital). Members: Hiroshi Ishiguro (Nagoya University Graduate School of Medicine), Kazuichi Okazaki (Kansai Medical University Kori Hospital), Terumi Kamisawa (Tokyo Metropolitan Komagome Hospital), Hiroyuki Miyagawa (Sapporo Kohsei Hospital), and Keisho Kataoka (Otsu City Hospital).

## The Japanese society of gastroenterology

President: Kazuhiko Koike (Kanto Central Hospital). Past President: Tooru Shimosegawa (South Miyagi Medical Center). Directors Responsible: Satoshi Mochida (Department of Gastroenterology and Hepatology, Saitama Medical University) and Nobuyuki Enomoto (First Department of Internal Medicine, Faculty of Medicine, University of Yamanashi).
